# Review of “ *mathematical models for neglected tropical diseases: essential tools for control and elimination, part A* ” edited by Roy M. Anderson and Maria-Gloria Basáñez

**DOI:** 10.1186/s13071-015-1126-5

**Published:** 2015-10-06

**Authors:** Christian Bottomley

**Affiliations:** MRC Tropical Epidemiology Group, London School of Hygiene & Tropical Medicine, Keppel Street, London, WC1E 7HT UK

## Book details

Advances in Parasitology Volume 87: *Mathematical Models for Neglected Tropical Diseases: Essential Tools for Control and Elimination, Part A.*

Academic Press; 2015.

417 pages. ISBN 978-0-12-803256-5

## Review

This volume in Advances in Parasitology, which summarises recent advances in mathematical modelling of Neglected Tropical Disease (NTDs), marks 30 years since the publication of a landmark review by Anderson and May on `Helminth infections of humans: mathematical models, population dynamics, and control’ in the same journal. The remit of the current review has been expanded to cover not just helminths, but NTDs in general, in response to the focus on NTDs since the World Health Organization’s 2020 Roadmap on NTDs, and the 2012 London declaration (http://unitingtocombatntds.org/). Most of the chapters review recent developments in the mathematical models of particular diseases (leprosy, human African trypanosomiasis, lymphatic filariasis, Chagas disease), although there are also chapters on the Allee effect, helminth egg count data, molecular epidemiology, and economic evaluation. So, what developments have there been in the intervening years since the original review?

Perhaps the most significant developments are related to improvements in computer power. One is the increasing use of micro-simulation models to model disease transmission. Unlike traditional models that track changes in the population, micro-simulation models track changes in the individual, and are therefore better able to account for differences between individuals - e.g., in their susceptibility to disease or compliance to treatment. Two good examples of the approach, reviewed in this volume, are the LYMFASIM model of lymphatic filariasis and the SIMCOLEP model of leprosy.

Algorithms for fitting models to data have also advanced considerably over the last couple of decades. As with micro-simulation, this has been facilitated by improvements in computing power. The process of fitting a model to data is useful for estimating parameters and validating the model. An excellent example is provided by Gambhir and colleagues who use a recently developed Bayesian algorithm to fit their model of lymphatic filariasis to data on microfilarial prevalence in different age groups. Despite improvements in methodologies, it can still be difficult to fit models to data, and examples like this are useful for improving best practice.

The way that models are used has also changed. Anderson and May used models to establish general conditions under which a disease is able to persist in a population. This type of analysis is still done (e.g. in the chapter on the Allee effect), but increasingly models are used to evaluate specific scenarios, which are often to do with different strategies of intervention. In the current volume, models are used, for example, to find out how long mass drug administration should be continued to eliminate filariasis; to evaluate whether leprosy can be controlled by giving chemoprophylaxis to contacts of a case; and to optimise the timing of vector control campaigns for Chagas disease.

Given that models are now frequently used to explore different scenarios for disease control or elimination, it is disappointing that they have had limited impact on policy (a point made by several authors in this volume). Perhaps this is because we are not providing policy makers with the information they need. The authors of the chapter on economic and financial evaluation make the case that we already have the tools to eliminate NTDs - they suggest, for instance, that lymphatic filariasis, onchocerciasis and trachoma could be eliminated from 14 Latin American and Caribbean countries at a cost of USD 128 million – and argue that models must consider costs, both of the intervention and the illness. This seems to be an important message if we want to use mathematical models to guide policy.

I learnt a lot from reading this volume. The reviews are accessible to the non-specialist, and as well as reviewing the modelling literature, many provide useful overviews of the relevant biology.

## Abbreviation

NTD: Neglected Tropical Disease

